# Brief Rewarming Blunts Hypothermia-Induced Alterations in Sensation, Motor Drive and Cognition

**DOI:** 10.3389/fphys.2016.00592

**Published:** 2016-12-01

**Authors:** Marius Brazaitis, Henrikas Paulauskas, Albertas Skurvydas, Henning Budde, Laura Daniuseviciute, Nerijus Eimantas

**Affiliations:** ^1^Institute of Sport Science and Innovations, Lithuanian Sports UniversityKaunas, Lithuania; ^2^Faculty of Human Sciences, Medical School HamburgHamburg, Germany; ^3^Sports Science Department, Reykjavik UniversityReykjavik, Iceland; ^4^Faculty of Social Sciences, Arts and Humanities, Kaunas University of TechnologyKaunas, Lithuania

**Keywords:** rewarming, cold stress, hypothermia, thermoregulation, motor and cognitive performance

## Abstract

**Background:** It is well known that cold exposure experienced during occupational or recreational activities may adversely affect motor, cognitive performance, and health. Most research has used prolonged passive external rewarming modalities and focused on the direct effects on the kinetics of physiological and psychological responses in hypothermic subjects. However, the brief whole body rewarming effects on physiological and psychological responses in parallel with functional consequences on cognitive and neurophysiological functions have not been investigated. This study explores these effects in 12 healthy young men.

**Methods:** Subjects (20 ± 1 years) participated in 4 randomized trials, which were designed to compare the effects of whole-body brief (5-min) rewarming in 37°C water with rewarming for the same duration in 24°C (air) thermoneutral environment in mildly hypothermic subjects. After each rewarming, indicators of neuromuscular function (reflexes, central activation ratio, electromyography of exercising muscle, and contractile properties of calf muscles) and cognitive function (attention, simple motor speed, and information processing speed) were assessed.

**Results:** Compared to rewarming in thermoneutral environment, after brief rewarming in 37°C water, significantly lower metabolic heat production (MHP) (206 ± 33.4 vs. 121.9 ± 24.3 W·m^2^, *P* < 0.01), heart rate (76 ± 16 vs. 60 ± 12 b·min^−1^, *P* < 0.01), cold strain (6.4 ± 3.1 vs. 5.3 ± 2.7, *P* < 0.01), improved thermal comfort and induced cessation of shivering were found. Electrically induced maximum torque amplitudes increased (P100, 102.8 ± 21.3 vs. 109.2 ± 17.5 Nm and PTT100, 83.1 ± 17.1 vs. 92.7 ± 16.0 Nm, *P* < 0.05), contraction half-relaxation time decreased (599.0 ± 53.8 vs. 589.0 ± 56.3 ms, *P* < 0.05), and M_max_-wave latency shortened (17.5 ± 2.2 vs. 15.6 ± 2.0 ms, *P* < 0.05) after 37°C water rewarming. Unlike rewarming in thermoneutral environment, 37°C water rewarming blunted the hypothermia-induced alterations in neural drive transmission (4.3 ± 0.5 vs. 3.4 ± 0.8 mV H-reflex and 4.9 ± 0.2 vs. 4.4 ± 0.4 mV V-wave, *P* < 0.05), which increased central fatigue during a 2-min maximum load (*P* < 0.05). Furthermore, only in brief warm water rewarming cerebral alterations were restored to the control level and it was indicated by shortened reaction times (*P* < 0.05).

**Conclusions:** Brief rewarming in warm water rather than the same duration rewarming in thermoneutral environment blunted the hypothermia-induced alterations for sensation, motor drive, and cognition, despite the fact that rectal and deep muscle temperature remained lowered.

## Introduction

Prolonged exposure to a severe cold environment causes marked whole-body cooling, defined as a decrease in core temperature, which can impair motor and cognitive performance by altering neural drive through central and peripheral failure (Giesbrecht et al., 1995; Rutkove et al., 1997; Cahill et al., 2011; Brazaitis et al., 2014b; Solianik et al., 2014). Even exposure to less severe cold, which does not lower core temperature markedly, may produce cognitive (Palinkas, 2001; Mäkinen et al., 2006; Muller et al., 2012) and physical (Drinkwater and Behm, 2007) decrements, which may adversely affect performance and health (Tanaka et al., 1993; Palinkas, 2001). Brief periodic rewarming breaks throughout the day are recommended for restoring or preventing impairments in physiological and psychological performance for people exposed to the cold in occupational or leisure activities (Ceron et al., 1995; Ozaki et al., 1998; Brown et al., 2012). For example, in a protocol involving 3 sets of alternating 20-min exposure to −25°C in an industrial freezer with 20-min rewarming at 30°C or rest at 10°C, the protocol involving rewarming had a more positive effect than rest at 10°C on body temperature, hand tremor, counting task performance, and thermal and pain sensations. However, the periodic rewarming at 30°C only partially prevented the large decrease in core temperature, and this rewarming protocol could not restore core temperature to the initial level (Ozaki et al., 1998). Most research has used prolonged passive external rewarming lasting from 30 min to several hours using a water bath, air inhalation, or forced air. These studies have focused on the direct effects of rewarming from hypothermia on the kinetics of physiological responses including body temperature (Hoskin et al., 1986; Kumar et al., 2015), cardiovascular parameters (Hayward et al., 1984; Savard et al., 1985), subjective sensation (Kumar et al., 2015), metabolic heat production (MHP), and heat gain (Goheen et al., 1997). The functional consequences of applying brief (5-min) whole-body rewarming on cognitive and neurophysiological functions in mildly hypothermic subjects have not been investigated.

In the laboratory setting, spontaneous endogenous rewarming from hypothermia or cold is performed by placing the subject in a thermoneutral ambient temperature of 21–25°C (with or without external cover), which is insufficient to inhibit cold and/or excite warm receptors and therefore induce a shift in thermogenesis until recovery (Romet, 1988; Giesbrecht and Bristow, 1992; Muller et al., 2012). In our study, we used a water temperature of 37°C (T_w_) for whole-body immersion of hypothermic subjects for 5 min to increase skin temperature (T_sk_) and to evoke alterations in peripheral thermo-TRP channels without allowing recovery of deep body temperature. Based on previous findings (Craig, 2003; Nakamura and Morrison, 2008; Vay et al., 2012) we reasoned that greater inhibition of cold-induced thermogenesis and cutaneous vasoconstriction in mildly hypothermic subjects are likely to occur during brief (5-min) whole-body rewarming at 37°C T_w_ rather than during rewarming for the same duration at thermoneutral 24°C (T_a_). Through the actions of warm-sensitive receptors during warm water immersion, the lamina I neurons carry temperature signals to the insular cortex, which is involved in discriminative temperature sensation (Craig, 2002, 2003); consequently, a hypothermic subject may feel warm and thermally comfortable despite a reduced rectal temperature (T_re_). We expected that a reduction in the physiological cold stress [assessed by cold strain index (CSI), (Moran et al., 1999)] and discomfort induced by T_w_ rewarming in hypothermic subjects may have a positive effect on cognitive performance because of the reduced amount of distraction (Teichner, 1958).

It has been proposed that ambient temperature and relative humidity can influence central drive through changes in T_sk_ or through integration of anticipatory and/or sensory feedback mechanisms (Cheung and Sleivert, 2004; Schlader et al., 2011; Levels et al., 2012). The spinal cord is a major site for modulation of motor drive (Racinais et al., 2008). Opposing responses of spinal modulation of neural drive have been observed for hyperthermia (Racinais et al., 2008; Brazaitis et al., 2015) and hypothermia (Brazaitis et al., 2014b; Solianik et al., 2014). Because this modulation relates partly to presynaptic inhibition or activation mediated by temperature-sensitive group III and IV afferents (Bigland-Ritchie et al., 1986; Duchateau et al., 2002; Avela et al., 2006), spinal modulation of neural drive is likely to be altered by brief T_w_ rewarming in mildly hypothermic subjects. If so, one would expect that increased conduction velocity induced by brief T_w_ rewarming in hypothermic subjects would require greater energetic resources for a sustained 2-min MVC by providing greater inhibitory feedback to central structures, which may contribute to greater central fatigue (Bigland-Ritchie and Woods, 1984).

Therefore, the main purpose of our study was to determinate whether brief rewarming in warm water is sufficient to negate hypothermia-induced central changes [i.e., central activation ratio (CAR), mean frequency (MnF), and root mean square (RMS) of the electromyography (EMG) signal, cognition, sensation, H-reflex, V-wave, and shift in thermogenesis] and peripheral changes (i.e., contractile properties of calf muscles and the M-wave). We hypothesized that brief (5-min) whole-body rewarming in T_w_ 37°C would blunt the hypothermia-induced alterations in thermoregulation, sensation, motor drive, and cognition, despite the lowered T_re_ and deep muscle temperature (T_mu_).

## Materials and methods

### Participants

Twelve healthy male volunteers participated in the study. Each participant read and signed a written informed consent form. Subjects with Raynaud's syndrome, asthma, neurological pathology, or conditions that could be worsened by exposure to cold water were excluded from this study. All participants were in self-reported good health, which was confirmed by medical history. The physical characteristics of the participants are presented in Table 1. Participants were moderately physically active (<3 times per week) and did not participate in any formal physical exercise or sports programme. The additional criteria for inclusion were age 18–30 years, being a non-smoker, and not taking medication or dietary supplements. All procedures were approved by LUHS Kaunas Region Biomedical Research Ethics Committee and were conducted according to the guidelines of the Declaration of Helsinki.

**Table 1:** **Physical characteristics of the subjects**.

Age, yr	20 ± 1
Height, cm	185.1 ± 6.8
Mass, kg	81.0 ± 13.2
Body mass index, kg/m^2^	23.7 ± 3.6
Body surface area, m^2^	2.0 ± 0.2
Body fat, %	15.4 ± 6.5
Mean subcutaneous fat, mm	12.6 ± 6.0

Values are means ± SD.

### Experimental design

#### Rationale for the experiment

**Figure 1:**
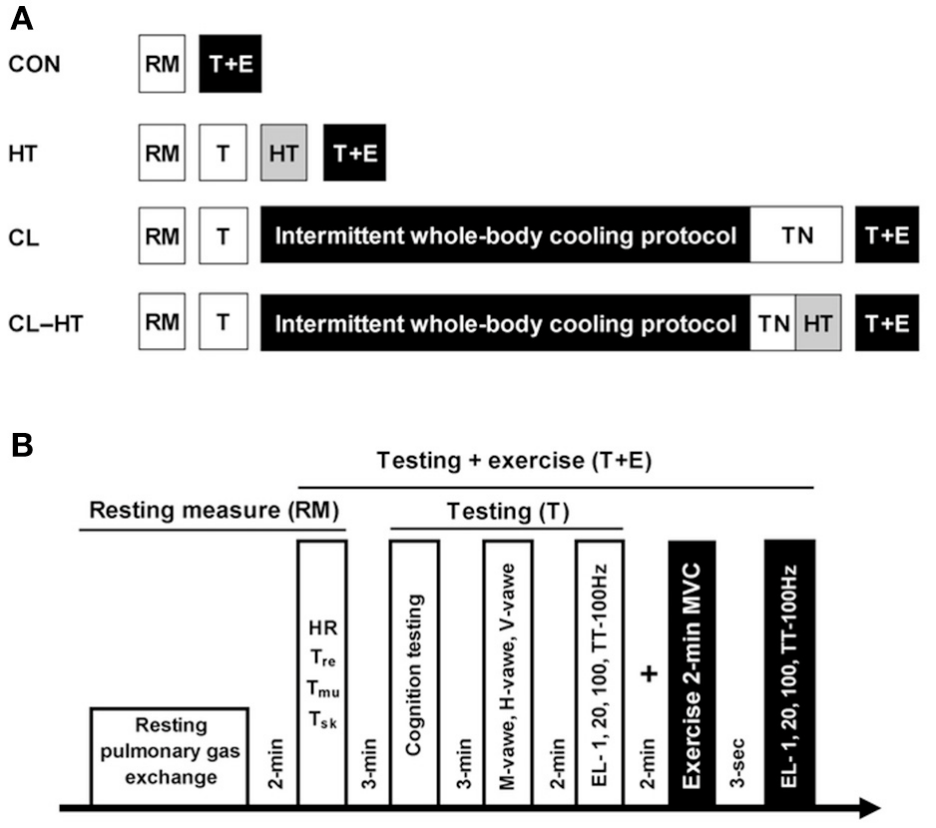
**Experimental design (A)**. RM, resting measure; HT, 5-min warming in T_w_; TN, thermoneutral environment (T_a_); T, testing; T+E, testing with the 2-min MVC exercise. Protocols for the experimental trials **(B)**. Heart rate (HR), body temperatures (rectal, T_re_; muscle, T_mu_; skin, T_sk_); cognition testing (simple, 2-choice and procedural reactions); sarcolemmal (M-wave), spinal (H-wave), and supraspinal (V-wave) excitability; effects of electrical stimulation [EL; i.e., 1, 20, 100 Hz and TT-100 Hz (a 250-ms test train of stimuli at 100 Hz)] on relaxed muscle indicate changes in peripheral capacity; maximum voluntary contraction (MVC) indicates changes in both central and peripheral capacity; central activation ratio was measured by TT-100 Hz superimposed on a 2-min MVC and indicates changes in central capacity.

The experiment was designed to induce whole-body mild hypothermia in young healthy men to induce alterations in thermoregulation, subjective sensation, motor drive, and cognition (Brazaitis et al., 2014b; Solianik et al., 2015b) and thereafter to compare the effects of whole-body brief (5-min) rewarming in warm water (T_w_ 37°C) (CL-HT trial) with those induced by rewarming for the same duration in a thermoneutral environment (T_a_ 24°C) (CL trial) on physiological and psychological variables (Figure 1A). To determine whether whole-body exposure to warm water under normothermia induces any physiological and psychological changes, normothermic subjects were immersed for 5 min in warm water (T_w_ 37°C) (HT trial), and the same variables were measured. In the control trial (CON), we assessed the same variables under normothermia without causing any changes in body temperatures (T_sk_, T_re_, T_mu_), and these variables were compared with those measured in the CL, CL-HT and HT trials.

#### Familiarization trial

The subjects were familiarized with the procedures over 3 days. One week before the experiment, participants were introduced to the experimental procedures and cognitive and neuromuscular testing, and informed of the known risks of the study. On each day, the subjects performed the cognitive test battery, and on day 3 they learned to achieve and maintain a maximum effort in plantar flexion for 3–4 s with a 250-ms stimulation train test at 100 Hz (TT-100 Hz) superimposed on an MVC, and their tolerance to electrical stimulation was assessed. Participants attended the laboratory at the same time of day within the time frame of 8:00–13:00 h to avoid circadian fluctuations in body temperature. They were instructed to sleep for a minimum 7–8 h on the night before the experiment; to refrain from alcohol, heavy exercise, and caffeine for at least 24 h; and to refrain from consuming any food for at least 12 h before arrival at the laboratory. To standardize the morning state of hydration, subjects were allowed to drink still water as desired until 60 min before the experiment. The study was performed at a room temperature of 24°C and 60% relative humidity.

#### CON trial

On arrival at the laboratory, the subject was asked to rest in a semi-recumbent posture for 10 min dressed in a T-shirt, swim shorts and socks. Resting pulmonary gas exchange was recorded for the next 20 min in the same semi-recumbent posture. Heart rate (HR), T_sk_, T_mu_, and T_re_ stabilization were assessed and recorded (Figure 1B). Within ~3 min after these resting measurements, the participant was seated at a table and performed the cognitive testing (see “*Cognitive performance*”). Upon completion of the cognitive test battery, the subject was positioned in the dynamometer chair, stimulating electrodes were placed over the tibial nerve, EMG electrodes were placed over the soleus (SOL) muscle of the right leg, and reflexes were assessed. After a 2-min rest, during which the electrodes were moved from over the tibial nerve to over the posterior calf muscle, the force-generating capacity of the posterior calf muscle was assessed by applying 1-s trains of electrical stimuli at P1, P20, PTT-100 Hz, and P100 Hz (see “*Force-generating capacity of posterior calf muscles*”). About 3 s was needed to change the stimulation frequency. After a 2-min rest, a 2-min isometric MVC was then performed. The PTT-100 stimulus was superimposed on the contraction at about 3 s and 120 s. The measurement at 3 s was repeated after the end of the 2-min MVC to measure involuntary force-generating capacity by imposing 1-s trains of electrical stimuli at P1, P20, PTT-100, and P100. In total, cognitive and neuromuscular testing procedures (T+E) took approximately 16 min (Figure 1B).

#### HT trial

Pulmonary gas exchange, HR, body temperature, cognitive performance, reflexes, and involuntary force-generating capacity were measured as described in the CON trial. After muscle testing, the subject began a brief (5-min) head-out immersion trial in warm water (T_w_ 37°C). During immersion, the subject remained in a semi-recumbent posture with his arms folded across the chest and the legs extended almost straight and together. Pulmonary gas exchange was recorded during the 5-min water immersion. Ratings of subjective perception were recorded at the end of the 5-min warming procedure. Within ~1 min after leaving the bath, the volunteer was towel dried and body temperatures were measured. After the end of the warming procedure, cognitive and neuromuscular testing was performed in the same order as before the warm water immersion. Thereafter, 2-min isometric MVC was assessed followed by assessment of involuntary force-generating capacity, as described above for the CON trial.

#### CL and CL-HT trials

Baseline measurements were made as described for the CON and HT trials. After muscle testing, the subject began the intermittent water-immersion cooling protocol as described in previous studies (Brazaitis et al., 2014a; Solianik et al., 2015a). Every 20 min during cooling, the subject was asked to step out of the bath and to rest for 10 min in the room environment and to then return to the water bath for the next 20 min of immersion. The water bath temperature was 14°C and the head-out immersion procedure was used. The procedure continued until T_re_ decreased to 35.5°C or until 170 min total (120 min maximum total immersion time), at which time the immersion ended regardless of T_re_. The exposure time until T_re_ achievement was recorded. During immersion and rest, the subject remained in a semi-recumbent posture with his arms folded across the chest and the legs extended almost straight and together. Ratings of subjective thermal and shivering sensation, and HR and T_re_ were recorded every 5 min throughout the cooling procedure. Pulmonary gas exchange was recorded only during each 20-min water immersion. Immediately after intermittent cooling, the subject was towel dried, and the body temperatures were measured.

In the CL-HT trial, within the 5 min needed to measure body temperatures and to change the bath water from cold to warm, the subject began brief (5-min) head-out rewarming in warm water (T_w_ 37°C) as described for the HT trial. In the CL trial, within the 5 min needed to measure body temperature, the subject was asked to rest in a semi-recumbent posture for 5-min rewarming at the thermoneutral T_a_ 24°C. In total, the time from the moment the subjects exited the cold water bath to the end of rewarming was 10 min and it was of the same duration in both CL and CL-HT trials. During these two brief rewarming procedures, pulmonary gas exchange and HR were recorded. Immediately after the T_w_ or T_a_ brief rewarming procedure, body temperatures were measured. Thereafter, cognitive and neuromuscular testing was performed in the same order as before the intermittent cold water immersion and ended with the 2-min isometric MVC followed by measurement of involuntary force-generating capacity, as described above for the CON trial.

### Experimental measurements

#### Anthropometric measurements

Body mass and body fat were measured, and body mass index was calculated. Body mass was measured as nude body mass on a body composition analyser (Tanita, TBF-300, Arlington Heights, IL, USA). Body surface area was estimated in m^2^ as = 128.1 × weight 0.44 × height 0.60 (Tikuisis et al., 2001). Skinfold thickness was calculated as the average thickness for 10 skinfold sites (chin, subscapular, chest, side, suprailium, abdomen, triceps, thigh, knee and calf) (McArdle et al., 1984) using a medical skinfold caliper (Seahan, SH5020, Masan, Korea).

#### Body temperature measurements

T_re_ was measured at rest and throughout each immersion procedure using a thermocouple (Rectal Probe; Ellab, Hvidovre, Denmark; accuracy ± 0.01°C) inserted to a depth of 12 cm past the anal sphincter. The rectal thermistor sensor was placed by each participant. The temperature measurements were taken before (all four trials) and after cooling (CL and CL-HT trials) or warm water (HT trial) immersion, and after brief rewarming (CL and CL-HT trials). T_sk_ was measured (Skin/Surface probe, DM852, Ellab; accuracy ± 0.01°C) with thermistors taped at three sites: back, thigh and forearm. The mean T_sk_ was calculated using the Burton (1935) equation: T_sk_ = 0.5_back_ + 0.36_thigh_ + 0.14_forearm_. T_mu_ was measured with a needle microprobe (MKA; Ellab) inserted at a depth of ~3.5 cm under the skin covering the lateral portion of the gastrocnemius in the right leg. For skin preparation before each intramuscular temperature measurement, the skin was shaved and disinfected before and after insertion of the microprobe using a cotton wool tampon soaked with medicinal alcohol. No local anesthesia was administered before insertion. After the first measurement, the insertion area was marked with a 0.5-cm-diameter circle to ensure that the same insertion point was used in later measurements before and after water immersion and rewarming in different trials.

#### CSI

As a rating of cold strain, the CSI was based on T_re_ and mean T_sk_, and was rated using a universal scale of 0 to 10 as follows: 1–2 (no or a little cold strain), 3–4 (low cold strain), 5–6 (moderate cold strain), 7–8 (high cold strain), and 9–10 (very high cold strain). The CSI was calculated as follows (Moran et al., 1999).

$$\begin{array}{l} \text{CSI }=\;6.67\;\left({\text{T}}_{\text{re}t}\;-\;{\text{T}}_{\text{re}0}\right)\;\times \;{\left(35\;-\;{\text{T}}_{\text{re}0}\right)}^{-1}\;\\ +\;3.33\;\left({\text{T}}_{\text{sk}t}\;-\;{\text{T}}_{\text{sk}0}\right)\;\times \;{\left(14\;-\;{\text{T}}_{\text{sk}0}\right)}^{-1} \end{array}$$

The measurements for CSI calculation were taken before (T_re0_, T_sk0_) and at the end (T_re*t*_, T_sk*t*_) of cold water immersion or at the end of brief rewarming; 14°C—water temperature; 35°C—T_re_ threshold.

#### Heart rate measurement

HR was measured throughout each trial with an HR monitor (S-625X, Polar Electro, Kempele, Finland). HR was recorded before (all four trials) and after cooling (CL and CL-HT trials) or warm water (HT trial) immersion and at the end of the brief rewarming (CL and CL-HT trials).

#### Spirometry

A mobile spirometry system (Oxycon Mobile, Jaeger/VIASYS Healthcare, Hoechberg, Germany) was used to measure pulmonary gas exchange at rest and during water immersion and rewarming. The system was used to monitor ventilatory parameters such as oxygen uptake (VO_2_) and CO_2_ production on a breath-by-breath basis. Calibration of this instrument was performed before recording according to the manufacturer's manual using the automatic volume- and gas-calibration functions. The gas analyser and delay time calibration were automatic as provided by the manufacturer: a calibration gas at 180 kPa (15.2% O_2_, 5.02% CO_2_, and 79.62% N_2_) was introduced to the Oxycon to attain gain, offset, and delay times within 1%. Oxygen consumption was recorded in 5-s increments. The first minute of data collected during each 20-min interval of intermittent cold water immersion and the first minute of data collected during 5-min warm water immersion or rewarming were not used in any calculations, as recommended by Tikuisis et al. (2000) because of reflex hyperventilation caused by cold or warm water immersion. MHP (in watts) was calculated from the respiratory gas exchange measurements of VO_2_ (in l/min) and the respiratory exchange ratio (RER = VCO_2_/VO_2_) according to the equation of Péronnet and Massicotte (1991) as MHP + (281.65 + 80.65 × RER) × VO_2_.

#### Measurement of perception

The method to measure subjective ratings for the whole body has been described elsewhere (Brazaitis et al., 2010, 2014b). Briefly, ratings of thermal sensation ranged from 1 (very cold) to 9 (very hot), with 5 being neutral. Thermal comfort sensation ranged from 1 (comfortable) to 5 (extremely uncomfortable), and the shivering/sweating sensation ranged from 1 (vigorously shivering) to 7 (heavily sweating), with 4 (not at all) being neutral. The rating of perception was reported by the participant every 5 min during immersion and at the end of T_a_ or T_w_ rewarming. The mean rating score value per immersion and rewarming was calculated.

#### Cognitive performance

To assess cognitive performance, the Automated Neuropsychological Assessment Metric (ANAM^4^, Norman, OK, USA) was administered (Reeves et al., 2010). A programmed cognitive test battery was used to assess changes in simple reaction, 2-choice reaction and procedural reaction times after the different immersion and/or rewarming procedures (see “Research Design”). All tasks were computer controlled, and the information was presented on the screen of a laptop (Samsung R538). All tests were performed in a quiet and semi-darkened laboratory with a laptop screen 40 cm in front of the participant. The participant was introduced to the test battery during the familiarization before the experiments began. The duration of the cognitive test battery was 3.0 ± 0.5 min and included the following tasks, which were randomized between experimental trials.

##### Simple reaction time test

This task measures simple visuomotor mental flexibility (Mäkinen et al., 2006; Kadota and Gomi, 2010). The test presents a simple stimulus with a symbol of “^*^” on the screen. The participant is instructed to press a specified response key as quickly as possible each time the stimulus is present. The accuracy for this task was 100% for all participants, and only response time was recorded.

##### 2-choice reaction time test

This task measures processing speed (Tzambazis and Stough, 2000) and alternating attention with a motor speed component (Kubicki et al., 2012). This test presents the user with a 2-choice symbol “^*^” or “o” on the display. The user is instructed to respond as quickly as possible by pressing the designated button for each stimulus as soon as the stimulus appears. The accuracy on this task was not less than 96% for all participants and did not differ significantly between trials, and only the response time was taken for analyses.

##### Procedural reaction time test

This test measures information processing speed, visuomotor reaction time, and attention (Churchill et al., 2016). A number (2, 3, 4, or 5) is presented on the display using a large dot matrix. The user is instructed to press 1 designated button for a “low” number (2 or 3) and another designated button for a “high” number (4 or 5). The accuracy on this task was not less than 94% for all participants and did not differ significantly between trials, and only the response time was taken for analyses.

#### Reflexes recordings

The participant was positioned in the dynamometer chair, stimulating electrodes were placed over the tibial nerve, EMG electrodes were placed over the SOL of the right leg, and the reflexes were assessed. After careful preparation of the skin (shaving, abrading, and cleaning with alcohol wipes) to obtain low impedance, bipolar Ag–AgCl surface bar electrodes (10-mm diameter, 20-mm center-to-center distance; DataLOG type no. P3X8 USB, Biometrics, Newport, UK) were used for EMG recording. For the SOL, the electrode was placed over the SOL ~13 cm above the calcaneus and below the muscle fibers of the gastrocnemius. The actual electrode position was marked, and the same recording site was used in the different trial measurements. The ground electrode was positioned on the tarsus of the same leg. EMG signals were recorded with an amplifier (gain 1000), with signal measurements using a third-order filter (18 dB/octave) bandwidth of 20–460 Hz. The analog signal was sampled and converted to digital form at a sampling frequency of 5 kHz. The EMG signal was telemetered to a receiver that contained a differential amplifier with an input impedance of 10 MΩ, the input noise level was less than 5 mV, and the common mode rejection ratio was higher than 96 dB. Before recording the EMG, we set the channel sensitivity at 3 V and excitation output at 4600 mV as recommended by the manufacturer. EMG files were stored simultaneously on the biometrics memory card and PC hardware, and dedicated software (Biometrics DataLOG) was used for data processing and analysis.

SOL H-reflexes, V-waves, and M-waves were evoked by 0.5-ms square-wave pulses stimulated by a cathode placed in the popliteal cavity and an anode placed distal to the patella over the posterior tibial nerve with an interelectrode distance of ~4 cm. The resting maximum H-reflex (H_max)_, which reflects the efficiency of transmission in Ia afferent motor neuron synapses, and maximum M-wave (M_max_), which reflects sarcolemmal excitability, were obtained by increasing the electrical intensity by 3 V every 10 s over the 30–150 V range. With increasing stimulation intensity, the H-reflex response initially increased progressively and then decreased and disappeared, whereas the M-wave achieved its maximum and remained stable. Thereafter, the participant was instructed to perform three brief MVCs of the plantar flexor muscles for 3–4 s with at least a 1-min rest between contractions. A superimposed stimulus (at M_max_ intensity) was evoked to obtain the V-wave (V_sup_). The peak-to-peak amplitude of the V-wave reflects the magnitude of the central descending neural drive to spinal motor neurons, although spinal factors such as motor neuron excitability and pre- or post-synaptic inhibition may also be involved (Aagaard et al., 2002). The M-wave amplitude was also used to normalize the amplitude of the reflex waves recorded (i.e., H_max_/M_max_ ratio). This was done to ensure that any changes in the evoked H_max_ and V_sup_ amplitudes reflected changes at the muscle fiber membrane or neuromuscular junction. The latencies of the electrically evoked action potentials were calculated from the stimulation artifact at the peak of the wave.

#### Force-generating capacity of posterior calf muscles

The force-generating capacity of the posterior calf muscles was assessed before and immediately after the 2-min MVC. The equipment and procedure for electrically stimulated torque have been described previously (Brazaitis et al., 2012). Briefly, muscle stimulation was applied using flexible surface electrodes (MARP Electronic, Krakow, Poland), covered with a thin layer of electrode gel (ECG–EEG Gel; Medigel, Modi'in, Israel), with one electrode (8 × 12 cm) placed transversely across the width of the proximal portion of the posterior calf just below the popliteal fossa, and the other electrode (8 × 8 cm) covering the distal portion of the muscle just below the muscle fibers of the gastrocnemius. A constant current electrical stimulator (MG 440; Medicor, Budapest, Hungary) was used to deliver 0.5-ms square-wave pulses at 150 V. Peak torques induced by a 1-s electrical stimulation at 1 Hz (P1; representing the properties of muscle excitation–contraction coupling), at 20 Hz (P20; representing the steep section of the force–frequency relationship curve) and at 100 Hz (P100; which is close to maximum force) were measured with a 3-s rest interval between electrical stimulation. The contraction and half-relaxation time (CT+HRT) was measured for the resting TT-100 Hz (250-ms test train of stimuli at 100 Hz) contractions. The CT+HRT was calculated as the time (in ms) taken for the torque to increase to the peak value and then to decrease to half of that value.

#### Exercise: 2-min MVC

The isometric torque of the ankle plantar flexion muscles was measured using an isokinetic dynamometer (System 4, Biodex Medical Systems, Shirley, NY) calibrated according to the manufacturer's service manual with a correction for gravity performed using the Biodex Advantage program (version 4.X). Participants were seated in the dynamometer chair with the trunk inclined at 45° with respect to the vertical and with hip, knee, and ankle joint angulations of 90°, 100° (full knee extension = 180°), and 90°, respectively. To assess 2-min sustained MVC endurance, the participant was instructed to achieve and maintain a maximum effort of ankle plantar flexion for 120 s. The trace was inspected visually to ensure that there were no artifact spikes at the start of the signal curve. The participant's arms were crossed on the chest with the hands grasping the trunk-supporting belt during all tests on the dynamometer. To help ensure a maximum effort, standard vocal encouragement was provided during the voluntary ankle plantar flexion trial by the same experienced investigator. During the 2-min MVC, the TT-100 Hz stimulus was superimposed on the contraction at 3 s (MVC-3s) and 120 s (MVC-120s) to assess the CAR of the plantar flexors (Brazaitis et al., 2015; Solianik et al., 2015a). The CAR was calculated using the following equation: CAR = MVC/(MVC + PTT100), where a CAR of 1 indicates complete activation, and a CAR <1 indicates central activation failure or inhibition. The percentage changes in CAR and fatigue index (FI; percentage decline in MVC torque) were calculated for MVC-3s and MVC-120s.

#### Muscles activity-generating capacity

The skin preparation, surface bar electrode, software, hardware, and measurement settings were the same as those described above for reflex recording. The EMG signals of the SOL were recorded by amplifiers (gain 1000) with signal measurements using a third-order filter bandwidth of 20–460 Hz. The analog signal was sampled and converted to the digital form at a sampling frequency of 5 kHz. EMG analysis was performed by calculating the RMS (as a measure of the EMG amplitude values) and MnF (as a measure of the EMG signal frequency content) for a 1000-ms epoch coinciding with a 1-s force interval just before each TT-100 Hz was superimposed on an MVC.

### Statistical analysis

The data were tested for normal distribution using the Kolmogorov–Smirnov test, and all data were found to be normally distributed. Descriptive data are presented as mean ± standard deviation (SD). Two-way ANOVA for repeated measures was used to determine the effects of experimental trials of two levels (CON vs. HT; CON vs. CL; CL vs. CL-HT; CON vs. CL-HT) and time of two levels [before (or MVC-3s) vs. after (or MVC-120s) 2-min MVC] on MVC, CAR, electrically induced muscle properties (P1, P20, P100, PTT-100, and CT+HRT), and RMS and MnF of the SOL EMG signal. Two-way ANOVA for repeated measures was also performed to study the effects of rewarming (two levels: CL vs. CL-HT; three levels: before cooling vs. after cooling vs. after rewarming) on body temperatures (T_re_, T_sk_, T_mu_), MHP and HR; and on the CSI (two levels: CL vs. CL-HT; two levels: after cooling vs. after rewarming). One-way ANOVA for repeated measures was used to determine the effects of the CON, HT, CL, and CL-HT experimental trials of four levels on resting body temperatures (T_re_, T_sk_, T_mu_), MHP and HR; and the effects of experimental trials of two levels as a between-trial factor (CON vs. HT; CON vs. CL; CL vs. CL-HT; CON vs. CL-HT) on the FI of MVC torque and changes in CAR, electrically induced muscle properties, and RMS and MnF of the RMS of SOL; and on the spinal and supraspinal reflex excitability and cognition. If significant effects were found, Sidak's *post hoc* adjustment was used for multiple comparisons across a set of conditions within each repeated-measure ANOVA. Statistical significance was defined as *P* < 0.05. Statistical power (SP, as a percentage) was calculated, and the partial eta squared (η_*p*_^2^) was estimated as a measure of the experimental trial effect size. The SP for a significant effect was >80%. The non-parametric Wilcoxon signed-rank test for two related samples was used to compare changes in subjective ratings of perceptions (thermal, comfort, and shivering sensations) after rewarming (vs. post-cooling) between CL and CL-HT trials. Statistical analyses were performed using IBM SPSS Statistics software (v. 22; IBM Corp., Armonk, NY).

## Results

For values measured before the experimental trials, there were no significant differences in body temperatures, HR, MHP, MVC, CAR, RMS, and MnF of the EMG signal, electrically induced muscle properties, cognition, and reflexes between conditions (CON vs. HT vs. CL vs. CL-HT) (*P* > 0.05; η_*p*_^2^ < 0.4, *SP* < 50%). Body temperatures, HR, MHP, CSI, and subjective sensations were not different between trials (CL vs. CL-HT) before and after whole-body cooling (*P* > 0.05; η_*p*_^2^ < 0.2, *SP* < 40%).

### Effects of CL-HT on body temperatures

In the CL trial, the time to cool the body from T_re_ 36.8 ± 0.3°C before cooling to 35.6 ± 0.7°C was 126.5 ± 48.4 min. In the CL-HT trial, the time to cool the body from T_re_ 36.9 ± 0.2°C before cooling to 35.5 ± 0.7°C was 123.1 ± 50.1 min (time effect; *P* < 0.001, η_*p*_^2^ > 0.9, *SP* > 99%; CL vs. CL-HT trial effect; *P* > 0.05; η_*p*_^2^ < 0.1, *SP* < 15%). T_re_ did not change 10 min after cooling in the thermoneutral environment in the CL trial (T_a_ 24°C) or after brief whole-body rewarming in warm water in the CL-HT trial (T_w_ 37°C) compared with the values measured immediately after cooling (time effect; *P* > 0.05, η_*p*_^2^ < 0.3, *SP* < 45%; CL vs. CL-HT trial effect; *P* > 0.05, η_*p*_^2^ < 0.2, *SP* < 30%) (Table 2). T_mu_ decreased after cooling and further still after rewarming (time effect; *P* < 0.01, η_*p*_^2^ > 0.8, *SP* > 99%). There were no differences between CL and CL-HT experimental trials (CL vs. CL-HT trial effect; *P* > 0.05, η_*p*_^2^ < 0.35, *SP* < 40%). T_sk_ decreased to a similar extent after whole-body cooling in the CL and CL-HT trials. Whole-body rewarming in T_a_ increased T_sk_ by about 6°C from the value immediately after cooling, whereas rewarming in T_w_ increased T_sk_ by about 16°C (time effect; *P* < 0.001, η_*p*_^2^ > 0.9, *SP* > 99%; CL vs. CL-HT trial effect; *P* < 0.001, η_*p*_^2^ > 0.9, *SP* > 99%), which restored T_sk_ to the normal pre-cooling value. T_mu_ and T_re_ did not change but T_sk_ increased by about 2°C during the HT trial.

**Table 2:** **Body temperature, heart rate (HR), metabolic heat production (MHP), cold strain index (CSI), and thermal, thermal comfort, and shivering sensations in the HT, CL, and CL-HT trials**.

	**HT**		**CL**			**CL-HT**		
	**Before**	**After**	**Before**	**After**	**10-min after CL**	**Before**	**After**	**After re-warming**
Rectal T, °C	36.9 ± 0.2	36.9 ± 0.2	36.8 ± 0.3	35.6 ± 0.7^*^	35.5 ± 0.8^*^	36.9 ± 0.2	35.5 ± 0.7^*^	35.4 ± 0.8^*^
Muscle T, °C	35.9 ± 0.4	36.1 ± 0.5	36.4 ± 0.5	31.6 ± 1.8^*^	30.1 ± 2.1^*^, ^§^	36.5 ± 0.4	30.7 ± 2.0^*^	30.2 ± 1.9^*^, ^§^
Skin T, °C	31.5 ± 0.6	34.6 ± 0.4^*^	32.2 ± 0.6	17.4 ± 0.7^*^	23.1 ± 1.7^*^, ^§^	32.2 ± 0.9	17.1 ± 0.6^*^	33.5 ± 0.6^§^^#^
HR, b min^−1^	58 ± 8	64 ± 13^*^	60 ± 10	78 ± 16^*^	76 ± 16^*^	61 ± 9	83 ± 15^*^	60 ± 12^§^^#^
MHP, W/m^2^	44.2 ± 5.3	52.3 ± 5.8^*^	43.6 ± 5.2	218.1 ± 55.2^*^	206.0 ± 33.4^*^	48.1 ± 13.7	220.1 ± 65.2^*^	121.9 ± 24.3^*^, ^§^, ^#^
CSI				7.2 ± 3.0	6.4 ± 3.1^§^		7.8 ± 2.3	5.3 ± 2.7^§^^#^
Thermal sensation		6.4 ± 0.8		3.3 ± 0.9	3.7 ± 1.1		3.4 ± 1.0	6.6 ± 0.5^§^^#^
Thermal comfort sensation		1.0 ± 0.0		1.5 ± 0.6	1.3 ± 0.5		1.5 ± 0.5	1.0 ± 0.0^§^^#^
Shivering sensation		4.0 ± 0.0		5.4 ± 0.6	5.8 ± 0.4		5.3 ± 0.6	4.1 ± 0.3^§^^#^

* , P < 0.05 compared to before;

§ , P < 0.05, compared to after;

# , P < 0.05, compared to CL. Values are means ± SD.

### Effects of CL-HT on HR and MHP

HR and MHP increased in the HT, CL and CL-HT trials (time effect; *P* < 0.01, η_*p*_^2^ > 0.85, *SP* > 95%) (Table 2). Rewarming in T_a_ did not changed HR and MHP values compared with immediately after cooling, whereas rewarming in T_w_ reduced HR to the value measured before cooling and lessened MHP by about 45% compared with immediately after cooling (CL vs. CL-HT trial effect; *P* < 0.01, η_*p*_^2^ > 0.8, *SP* > 90%).

### Effects of CL-HT on CSI and subjective ratings of sensation

Whole-body cooling caused high cold strain in both the CL and CL-HT trials (Table 2). The CSI decreased more after T_w_ rewarming than after T_a_ rewarming (CL vs. CL-HT trial effect; *P* < 0.01, η_*p*_^2^ > 0.8, *SP* > 90%). The subjects felt warmer and experienced greater thermal comfort, and reported that their shivering stopped during T_w_ rewarming. By contrast, no changes in these aspects of sensation were reported by the subjects during T_a_ rewarming.

### Effects of CL-HT on spinal and supraspinal reflex excitability

Whole-body cooling (CL trial) increased the amplitude of H_max_ and V_sup_, H_max_/M_max_ ratio, V_sup_/M_max_ ratio, and the latency times of H_max_, V_sup_, and M_max_ responses (time effect; *P* < 0.01, η_*p*_^2^ > 0.8, *SP* > 95%) (**Table 4**). All of these reflex parameters decreased significantly after T_w_ rewarming (CL vs. CL-HT trial effect; *P* < 0.05, η_*p*_^2^ > 0.8, *SP* > 90%), although none reached the CON values. Spinal and supraspinal reflex excitability did not change during the HT trial.

### Effects of CL-HT on skeletal muscle torque-generating capacity

The 2-min MVC significantly decreased the electrically induced muscle torque (P1, P20, P100, and PTT-100) and significantly increased the CT+HRT of PTT100 in all four experimental trials (time effect; *P* < 0.01, η_*p*_^2^ > 0.8, *SP* > 95%) (Table 3). Intermittent whole-body cooling decreased P20, P100, and PTT100 torque and increased CT+HRT of PTT100 measured before exercise compared with the normothermic condition (*P* < 0.01, η_*p*_^2^ > 0.8, *SP* > 90%). Brief rewarming in T_w_ increased P100 and PTT100 torque and decreased CT+HRT of PTT100 measured before exercise compared with the values in the CL trial (*P* < 0.05, η_*p*_^2^ > 0.7, *SP* > 80%). However, these changes remained significantly different from those measured in the CON trial. The changes in P20 torque and CT+HRT of PTT100 from before to after the 2-min MVC were significantly smaller, and the change in P100 torque was significantly larger in the CL trial than in the CON trial. Brief rewarming in T_w_ decreased only the size of the change in P100 torque compared with the CL trial (*P* < 0.05, η_*p*_^2^ > 0.7, *SP* > 80%). The percentage change in P1 and PTT100 did not differ significantly between trials. The electrically induced muscle properties were not affected by the HT trial.

**Table 3:** **Electrically induced muscle properties before and after the 2-min MVC in the control trial (CON) and experimental trials after 5-min body heating (HT), prolonged body cooling (CL), and prolonged body cooling followed by 5-min of rewarming (CL-HT)**.

	**CON**		**HT**		**CL**		**CL-HT**	
	**Before**	**After**	**Before**	**After**	**Before**	**After**	**Before**	**After**
P1, Nm	24.2 ± 5.6	19.3 ± 3.1^*^	23.0 ± 5.8	19.6 ± 7.3^*^	24.1 ± 5.8	19.5 ± 3.8^*^	25.7 ± 6.2	20.0 ± 3.4^*^
Δ, %		−24.8 ± 19.3		−23.6 ± 32.4		−23.5 ± 20.1		−28.6 ± 23.4
P20, Nm	114.8 ± 19.3	82.4 ± 11.4^*^	112.8 ± 20.8	78.5 ± 15.5^*^	105.9 ± 22.9^‡^	82.4 ± 16.4^*^	109.4 ± 18.7^‡^	87.5 ± 16.0^*^, ^#^
Δ, %		−39.1 ± 13.2		−32.7 ± 7.2		−28.2 ± 10.9^‡^		−25.6 ± 11.0^‡^
P100, Nm	124.8 ± 20.3	97.5 ± 15.4^*^	123.1 ± 18.9	92.7 ± 17.2^*^	102.8 ± 21.3^‡^	72.9 ± 16.2^*^,^‡^	109.2 ± 17.5^‡^, ^#^	83.5 ± 15.4^*^,^‡^, ^#^
Δ, %		−29.3 ± 15.8		−22.6 ± 10.2		−41.7 ± 13.3^‡^		−31.9 ± 14.3^#^
PTT100, Nm	109.4 ± 17.9	89.5 ± 12.4^*^	108.8 ± 15.5	82.6 ± 13.9^*^	83.1 ± 17.1^‡^	66.5 ± 13.6^*^,^‡^	92.7 ± 16.0^‡^, ^#^	76.7 ± 12.8^*^,^‡^, ^#^
Δ, %		−23.0 ± 14.1		−19.9 ± 8.4		−25.5 ± 8.5		−21.0 ± 11.7
CT+HRT, ms	441.0 ± 13.7	562.0 ± 54.5^*^	431.0 ± 20.6	547.0 ± 32.9^*^	504.0 ± 29.5^‡^	599.0 ± 53.8^*^,^‡^	484.0 ± 30.1^‡^, ^#^	589.0 ± 56.3^*^,^‡^
Δ, %		26.3 ± 6.8		24.1 ± 5.5		15.3 ± 7.9^‡^		17.2 ± 8.4^‡^

* , P < 0.05 compared to before;

‡ , P < 0.05, compared to CON;

# , P < 0.05, compared to CL. Values are means ± SD. Δ 2-min MVC induced alteration in percent.

### Effects of CL-HT on MVC and CAR

**Figure 2:**
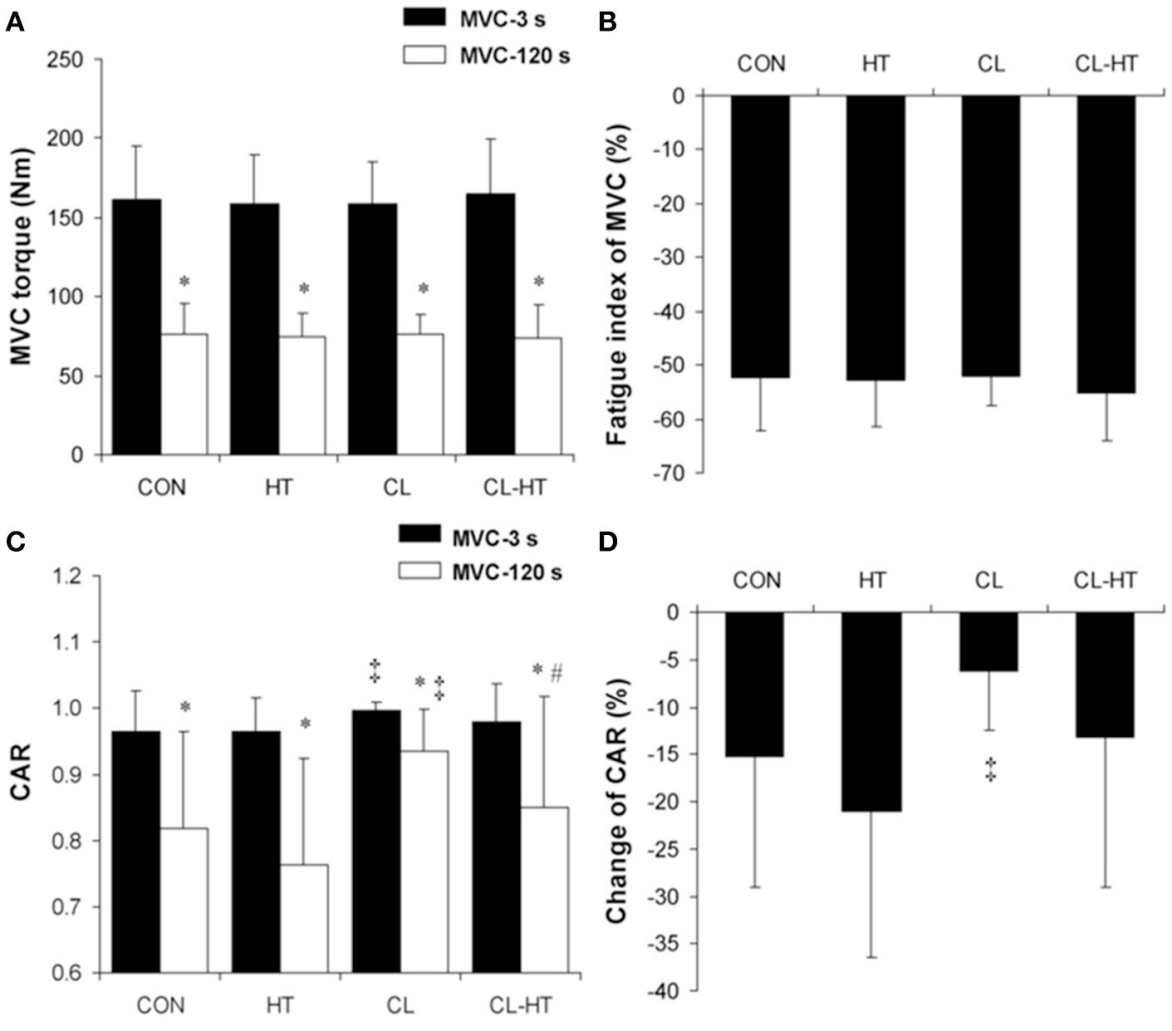
**Changes in maximum voluntary contraction (MVC) torque (A)**, fatigue index of MVC torque **(B)**, central activation ratio (CAR) **(C)**, and percentage change in CAR **(D)** during the 2-min MVC in the control trial (CON) and trials with 5-min body heating (HT), prolonged body cooling (CL), and prolonged body cooling with 5-min of rewarming (CL-HT). ^*^P < 0.05 compared with MVC-3s; ^‡^*P* < 0.05, compared with CON; ^#^*P* < 0.05, compared with CL. Values are mean ± SD.

The MVC torque (Figure 2A) and CAR (Figure 2C) decreased over the exercise time (2-min MVC) in all four experimental trials (time effect; *P* < 0.001, η_*p*_^2^ > 0.9, *SP* > 99%). The FI of MVC did not differ significantly between the trials (Figure 2B). The percentage change in CAR was smaller in the CL trial than in the CON trial (*P* < 0.01, η_*p*_^2^ > 0.8, *SP* > 95%) (Figure 2D). Rewarming in T_w_ (CL-HT) caused a greater increase in CAR over the exercise time compared with the change in the CL trial (*P* < 0.05, η_*p*_^2^ > 0.75, *SP* > 90%). The change in CAR did not differ between the CON and CL-HT trials, or between the CON and HT trials.

### Effects of CL-HT on skeletal muscle EMG

**Figure 3:**
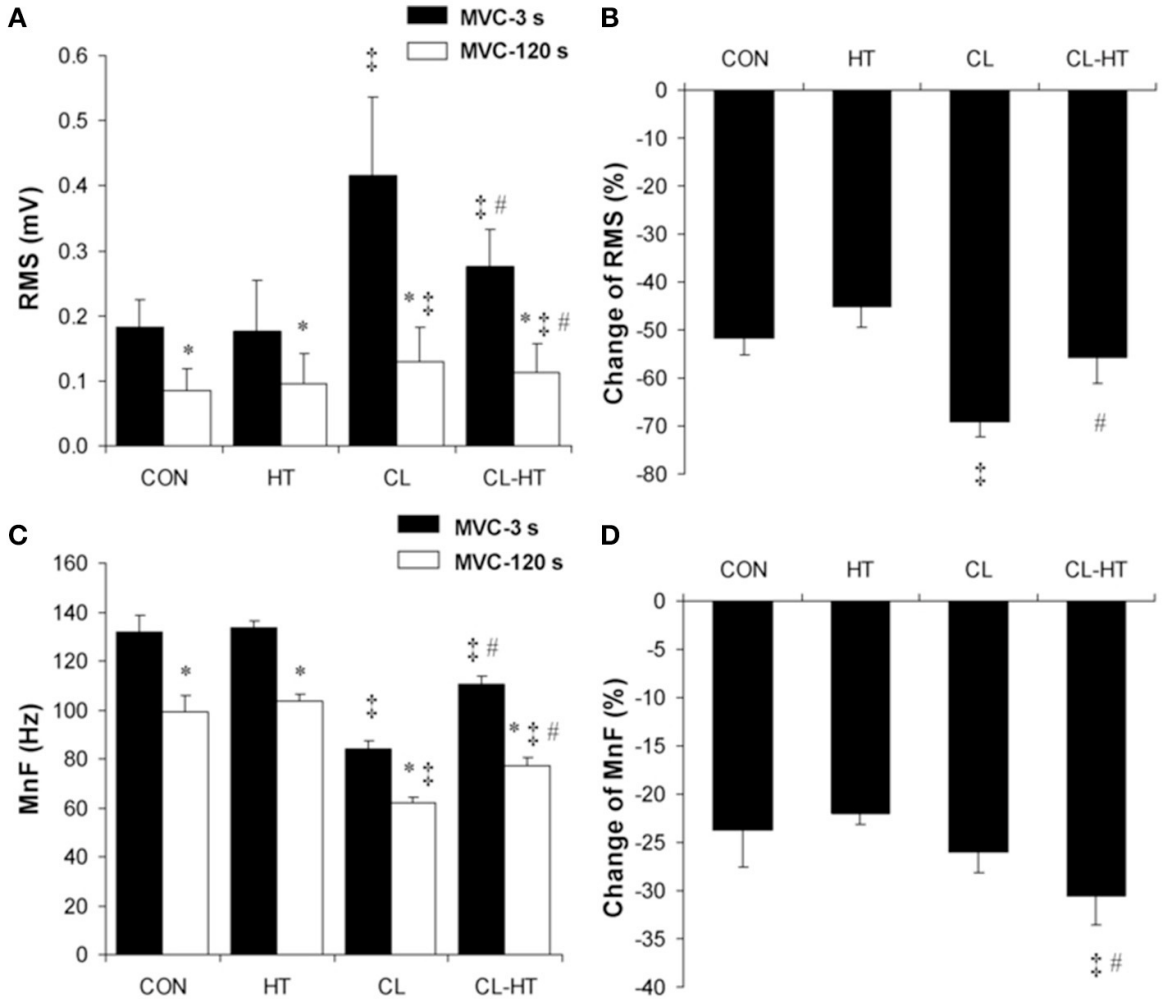
**Changes in the root mean square (RMS) values (A)**, percentage changes in RMS **(B)**, mean frequency (MnF) values **(C)**, and percentage changes in MnF **(D)** of the surface EMG signal of soleus muscle during the 2-min MVC in the control trial (CON) and trials with 5-min brief body heating (HT), prolonged body cooling (CL), and prolonged body cooling followed by 5-min rewarming (CL-HT). ^*^P < 0.05 compared with MVC-3s; ^‡^*P* < 0.05, compared with CON; ^#^*P* < 0.05, compared with CL. Values are mean ± SD.

The RMS (Figure 3A) and MnF (Figure 3C) of the surface EMG signal of SOL decreased over the exercise time (2-min MVC) in all four experimental trials (time effect; *P* < 0.001, η_*p*_^2^ > 0.9, *SP* > 99%). Body cooling (CL) significantly increased the SOL RMS and decreased the SOL MnF measured at MVC-3s and MVC-120s in the 2-min MVC (CL vs. CON trial effect; *P* < 0.001, η_*p*_^2^ > 0.9, *SP* > 99%). In contrast to CL, brief rewarming in T_w_ decreased the SOL RMS and increased the SOL MnF (*P* < 0.05, η_*p*_^2^ > 0.8, *SP* > 90%); however, neither of these EMG parameters reached the CON values. The change in the SOL RMS was greater in CL than in CON (*P* < 0.01, η_*p*_^2^ > 0.85, *SP* > 99%) (Figure 3B), whereas the change in the SOL MnF did not differ between the CL and CON trials (Figure 3D). Interestingly, brief rewarming in T_w_ decreased the change in the SOL RMS and increased the change in the SOL MnF over the exercise time compared with the change in the CL trial (*P* < 0.01, η_*p*_^2^ > 0.8, *SP* > 95%). Moreover, brief rewarming in T_w_ caused a similar change in SOL RMS during the exercise compared with the CON trial. The HT trial caused no changes in the surface EMG parameters measured in SOL.

### Effects of CL-HT on cognitive performance

**Figure 4:**
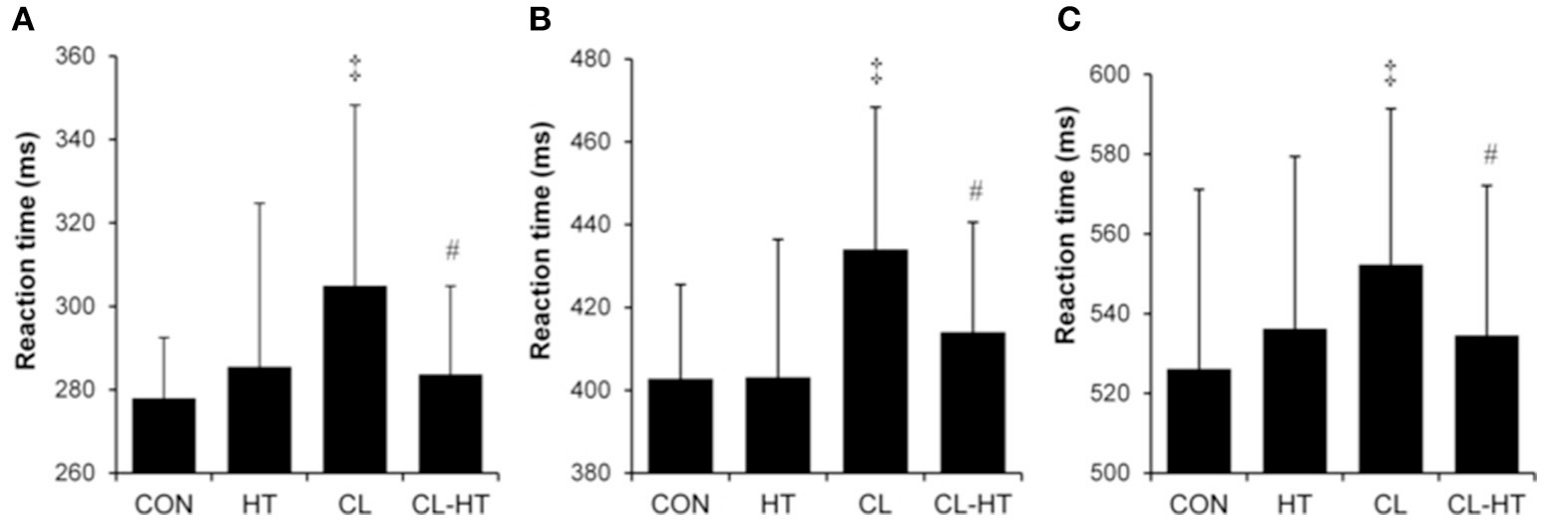
**Simple (A)**, 2-choice **(B)**, and procedural **(C)** reaction times in the control trial (CON) and experimental trials after 5-min body heating (HT), prolonged body cooling (CL), and prolonged body cooling followed by 5-min rewarming (CL-HT). ^‡^*P* < 0.05, compared with CON; ^#^*P* < 0.05, compared with CL. Values are mean ± SD.

Simple (Figure 4A), 2-choice (Figure 4B) and procedural (Figure 4C) reaction times increased significantly (i.e., slower reactions) in the CL trial compared with the CON trial (*P* < 0.05, η_*p*_^2^ > 0.75, *SP* > 90%). Brief rewarming in T_w_ decreased the reaction time (faster reaction) (CL vs. CL-HT trial effect; *P* < 0.05, η_*p*_^2^ > 0.7, *SP* > 80%), and the results for all three tests did not differ significantly from those in the CON trial. The HT trial caused no changes in reaction time. The accuracy (errors) for simple reaction task was 100%, for 2-choise reaction task > 96%, and for procedural reaction task > 94% for all participants, respectively, and did not differ significantly between the four trials.

## Discussion

To our knowledge, this is the first study to investigate the effects of brief rewarming on thermoregulation, sensation, motor drive, and cognition in mildly hypothermic subjects. The unique finding of our study is that, in mildly hypothermic subjects, brief (5-min) whole-body rewarming at 37°C T_w_ rather than rewarming for the same duration at 24°C T_a_ induced a thermosensory excitation shift by inhibiting body heat production (decreased MHP, HR, and cessation of shivering). In parallel, despite the reduced deep T_mu_ and T_re_, the subjects reported sensations of warmth and thermal comfort, which were accompanied by reduced CSI. Brief T_w_ rewarming increased the electrically induced maximum torque amplitude of TTP100 and P100, and decreased CT+HRT, and these changes were accompanied by shortened M_max_-wave latency. Brief rewarming in warm water blunted the hypothermia-induced alterations in the transmission of the neural drive at the spinal and neuromuscular junction levels. Thus, during prolonged contraction (2-min MVC), the additional failures at the supraspinal level seemed to impair motor drive to a greater extent in the CL-HT than in the CL trial. Cerebral alterations caused by hypothermia were confirmed by the increased reaction times in simple and more complex tasks, which were restored to the CON levels by brief rewarming in T_w_.

### Brief T_w_ rewarming inhibits heat production, induces sensations of warmth and thermal comfort in hypothermic subjects

It has been shown that the skin plays a large modulatory role in thermoregulation and thermal perception in the cold, despite the fact that core (i.e., tympanic) temperature is low (Benzinger, 1963; Cabanac et al., 1987). In agreement with these observations, we found that brief whole-body T_w_ rewarming rather than T_a_ rewarming of the same duration in mildly hypothermic male subjects induced a thermosensory excitation shift. This thermoregulatory shift is indicated in our study by the significant decrease in HR and MHP, and cessation of shivering reported by the subjects. This shift seems to have been affected solely by the increase in T_sk_ at ~16°C (before the cooling of T_sk_ ~33°C was reached) during the brief T_w_ rewarming phase (Table 2). However, these changes do not appear to have been affected by the increase of ~6°C during the brief T_a_ rewarming phase despite the similar decline and no further change in core temperature observed in both rewarming phase trials (CL-HT and CL). The brief rewarming in T_a_ of ~24°C increased the mean T_sk_ from 17.4°C to 23.1°C, and this change probably corresponded to an innocuous cool thermal threshold, which may have been too low to activate the predominant TRPM8 innocuous cool channel to shift and/or excite potential TRPV3 innocuous warm channels (Craig, 2002; Vay et al., 2012). The findings of our study suggest that, the 5-min T_w_ (peripheral) rewarming was sufficient to inhibit shivering and to reduce HR and MHP activities despite the reduced T_re_.

We observed that whole-body immersion in cold water at 14°C increased the CSI, subjective thermal sensation, and thermal discomfort sensation evaluated throughout the cooling period. TRPA1 and/or TRPM8 channels are activated by 14°C cold water, and this information is conveyed to the insular cortex via thin myelinated Aδ fibers to temperature-sensing lamina-1 neurons with a relay in the posterolateral thalamus (specifically, the posterior part of the ventromedial nucleus) or with two relays (in the parabrachial nucleus and the basal part of the ventromedial nucleus of the thalamus) (Craig, 2002, 2003). These two branches of the spino-thalamo-cortical pathway are involved in discriminative temperature sensation but do not play a significant role in the triggering of involuntary thermoregulatory responses (Craig, 2002). This means that psychological (thermal perception for making decisions) and physiological (thermoregulatory to defend body temperature) responses to the same temperature input probably act independently. Our study results are consistent with these observations in that we found that the thermal discomfort sensation during cooling increased by only 0.5 point (i.e., ranged from “comfortable” to “a little uncomfortable”) and thermal sensation ranged from “slightly cool” to “cool.” These slight changes suggest that prolonged immersion in cold water caused only a low to moderate psychological stress in these subjects, whereas the physiological CSI increased to “high cold strain.” In such conditions, brief rewarming in 37°C T_w_ rather than rewarming in 24°C T_a_ may excite TRPV3 channels and temperature signal sensing. This should induce a shift from cold-activated myelinated Aδ sensing fibers to warm-activated unmyelinated C sensing fibers and then to activate lamina-1 neurons and finally the insular cortex; consequently, the subjects felt warm and thermal comfort (Table 2).

### Brief T_w_ rewarming modulates reflexes in hypothermic subjects

The CL trial increased the excitability (amplitude and latency) of spinal (H_max_-reflex) and supraspinal (V_sup_-wave) reflexes. Among the measures of sarcolemmal excitability (M_max_-wave), cooling significantly increased only latency time. As reported by Drinkwater and Behm (2007), a lower muscle temperature may be needed to affect M_max_-wave amplitude than the T_mu_ found in this study (~22°C vs. ~30°C, respectively). These data agree with those of Brazaitis et al. (2014b) and Solianik et al. (2014), who showed that the H−, V−, and M-waves, and H_max_/M_max_ and V_sup_/M_max_ were facilitated after cold exposure.

In our study, we examined whether brief rewarming in the CL-HT trial would suppress excitability of reflexes in SOL compared with the CL trial. We found that, immediately after cooling, the T_mu_ at ~3.5 cm depth reached 31.6° and 30.7°C in the CL and CL-HT trials, respectively. Brief rewarming in both trials caused further decreases of about 1.5° and 0.5°C in T_mu_, respectively. Although we did not measure T_mu_ at different muscle depths, the increased T_sk_ to the value before cooling in the CL-HT trial indicating a withdrawal of vasoconstriction in the skin (Romanovsky, 2014) and greater heat exchange between the body and warm bath water (whose heat transfer is 25 times greater than air Toner and McArdle, 1996). This may have increased temperature in the superficial skin layer and possibly in nearby muscles without increasing T_re_ and deep T_mu_. As a result, the amplitude and latency of H_max_-reflex and V_sup_-wave were lower in the CL-HT than in the CL trial. It has been suggested that this spinal and/or supraspinal modulation is related to presynaptic inhibition, in which the decline in transmission from Ia afferent stimulation in α-motoneuron excitation may result from presynaptic inhibition mediated by temperature-sensitive group III and IV afferents (Bigland-Ritchie et al., 1986; Duchateau et al., 2002; Avela et al., 2006).

### Brief T_w_ rewarming improves electrophysiological function of exercising muscle in hypothermic subjects

In parallel with the changes in excitability of spinal and/or supraspinal reflexes, whole-body cooling in the CL trial decreased the MnF and increased the amplitude RMS of MVC in the surface EMG in SOL. These changes may reflect slowed opening and closing of the Na^+^ channels in the nerve and muscle membranes (Hodgkin and Katz, 1949). Specifically, the slower depolarization, which translates into a reduced axonal conduction velocity, and repolarization produce a longer channel-open time and, therefore, a longer duration response. The movement of additional Na^+^ ions into the cell during the prolonged opening leads to greater depolarization and response amplitude (Rutkove, 2001). We also observed a suppression effect of brief rewarming in the CL-HT compared with the CL trial that was sufficient to increase the MnF and decrease the RMS. This supports the idea of opposing mechanisms of excitation of the motor neuron pool caused by heating (Rutkove, 2001), which in our study seemed to have been modified by changes in peripheral rather than central temperature input.

### Brief T_w_ rewarming improves contractile properties of calf muscles in hypothermic subjects

In the CL trial, much of the decreases in electrically induced maximum torque amplitude of P20, TTP100, and P100, and of the increase in CT+HRT are consistent with peripheral muscle effects, including slowing of sarcoplasmic reticulum (SR) ATPase activity and ATP utilization (Ferretti, 1992), slowed Ca^2+^ release and uptake rate from the SR (Kössler et al., 1986), delayed cross-bridge formation, and detachment (Vanggaard, 1975; Faulkner et al., 1990), and decreased actomyosin sensitivity to Ca^2+^ (Sweitzer and Moss, 1990). It is postulated that, during and after cold exposure, M_max_-wave latency time increases because of the reduced ATP hydrolysis rate, which causes slowing of sarcolemmal action pumps through reduced activity of Na/K-ATPase (Drinkwater and Behm, 2007).

The novel contribution of the present study was the use of brief T_w_ rewarming after whole-body cooling (CL-HT vs. CL), during which TTP100 and P100 torque increased and CT+HRT decreased. These changes were accompanied by shortened M_max_-wave latency (Table 4), which generally corresponds to the increased muscle fiber conduction velocity, probably because of a temperature-mediated effect on voltage-gated Na^+^ channels (Rutkove, 2001), alongside the elevated ATP turnover (Gray et al., 2006). Specifically, the opening and closing of these channels accelerate, which allows less Na^+^ to enter cells and leads to a more rapid onset of depolarization (Rutkove et al., 1997). In the CL trial, P100 torque decreased more than P20 torque. This might be explained by the rise of [K^+^]o and [Na^+^]i during the high-frequency stimulation and may indicate that the Na^+^/K^+^ pump capacity is limited by the decrease in T_mu_, particularly in the T-tubules whose pump density is lower than that in the sarcolemma (Fitts, 1994). In the CL trial, potential depletion of ATP immediately after the 2-min MVC (Edwards et al., 1972) may have decreased P100 torque even more than that for P20 torque (high-frequency fatigue). Interestingly, the P20/P100 ratio was partly restored by brief T_w_ rewarming in the CL-HT trial (Table 3).

**Table 4:** **Spinal and supraspinal reflex excitability in the control trial (CON) and experimental trials after 5-min body heating (HT), prolonged body cooling (CL), and prolonged body cooling followed by 5-min of rewarming (CL-HT)**.

**Amplitude (mV)**					**Latency (ms)**			
**CON**	**HT**	**CL**	**CL-HT**		**CON**	**HT**	**CL**	**CL-HT**
2.8 ± 1.1	2.6 ± 1.3	4.3 ± 0.5^‡^	3.4 ± 0.8^‡^, ^#^	H_max_	40.0 ± 2.3	40.8 ± 2.4	46.6 ± 2.7^‡^	44.9 ± 2.3^‡^, ^#^
3.9 ± 0.7	3.7 ± 0.7	4.9 ± 0.2^‡^	4.4 ± 0.4^‡^, ^#^	V_sup_	37.7 ± 2.1	37.6 ± 2.0	45.2 ± 3.8^‡^	42.5 ± 2.8^‡^, ^#^
4.3 ± 0.7	3.9 ± 0.7	4.2 ± 0.6	4.2 ± 0.7	M_max_	12.3 ± 1.4	12.6 ± 1.6	17.5 ± 2.2^‡^	15.6 ± 2.0^‡^, ^#^
0.7 ± 0.3	0.7 ± 0.3	1.1 ± 0.3^‡^	0.8 ± 0.1^‡^, ^#^	H_max_/M_max_				
1.0 ± 0.5	0.9 ± 0.3	1.2 ± 0.3^‡^	1.1 ± 0.4^‡^, ^#^	V_sup_/M_max_				

‡ , P < 0.05 compared to control;

# , P < 0.05, compared to CL. Values are means ± SD.

### Brief T_w_ rewarming increases central fatigue in hypothermic subjects

We found that the maximum voluntary activation (CAR) of exercising muscles (MVC-3s) increased after whole-body cooling in the CL trial and was restored to the CON level by the brief T_w_ rewarming in the CL-HT trial (Figure 2C). These changes in CAR may be linked to modification of the transmission of motor drive in any of the stages from cortical activity to sarcolemma depolarization. This modification may have maintained MVC torque, which was similar in all four trials (Figure 2A), despite the fact that the electrically induced contractile properties of the muscle were impaired by whole-body cooling and were partly restored by brief T_w_ rewarming (Table 3). The sustained 2-min MVC combined with the superimposed electrical stimulation (TT-100 Hz) allowed us to provoke and distinguish central from peripheral fatigue of the ankle plantar flexors (Bernecke et al., 2015). Consistent with previous reports (Cahill et al., 2011; Solianik et al., 2015a), our data suggest that hypothermia offset central failure during sustained MVC (Figure 2C). Moreover, the more rapid muscle energetics (indicated by shortened M-wave latency, faster CT-HRT, increased MnF, and decreased RMS) and accelerated metabolite accumulation with brief T_w_ rewarming (vs. CL) in fatiguing muscle may have contributed to greater central fatigue by providing inhibitory feedback to central structures (Bigland-Ritchie and Woods, 1984), similar to that related to impaired excitability of spinal and/or supraspinal reflexes. It was previously suggested that, during sustained maximum contraction, the magnitude of voluntary activation of exercising muscle is inversely proportional to whole-body temperature within a moderate range from normal (Todd et al., 2005; Cahill et al., 2011). Our data add a specified functional perspective to this observation by showing that central voluntary activation of exercising muscle can be modified by brief T_w_ rewarming after whole-body hypothermia without affecting T_re_ and deep T_mu_.

### Brief T_w_ rewarming restores attention in hypothermic subjects

Our data suggest that these subjects experienced significant alterations in the synaptic transmission of neural influx at both the peripheral and spinal levels, and raise questions about similar alterations at the supraspinal level. We assessed cognitive function as an indicator of changes at the cortical level. Consistent with previous research (Mäkinen et al., 2006; Muller et al., 2012), we found that acute whole-body cold stress (CL vs. CON) caused slower reaction times in simple and more complex tasks (Figure 4). These findings are consistent with the distraction hypothesis, which suggests that discomfort (increased shivering, MHP, CSI, HR, subjective thermal sensations, and thermal discomfort) caused by cold stress causes a shift of attention from the primary task and leads to impaired performance (Teichner, 1958). A significant slowing in nerve conduction velocity (Table 4) could also explain the slower reaction in the CL trial. Interestingly, in the study by Muller et al. (2012) cognitive dysfunction persisted into the recovery period (60-min rest in 25°C air) after removal from the cold (120-min rest in 10°C air). By contrast, in our study, brief (5-min) whole-body T_w_ rewarming in the CL-HT trial blunted the hypothermia-induced alterations in simple, 2-choice, and procedural reaction times and allowed these parameters to return to the CON values (Figure 4). The restored reaction times in the CL-HT trial are also consistent with improved nerve conduction velocity (Table 4).

## Limitations

The findings of this study suggest that brief T_w_ rewarming in hypothermic subjects improved cognition, neural drive, and contractile properties of calf muscles, and reduced physiological and psychological cold stress. In our study T_re_ was measured as a marker of body core temperature. The temperature of this location is known to be relatively delayed during thermal transients. Thus, rectal temperature may not truly be indicative of the core temperature input received by the brain and/or other body locations. Deep body temperatures were not restored by brief T_w_ rewarming to normal, and we can only speculate on the later post-rewarming effects on thermoregulation, motor drive, cognition, and sensation. Moreover, the thermal conditions in the CL trials are not steady state as the testing environment is different from the prior intervention environment. To rewarm the mildly hypothermic subjects, we used brief (5-min) whole-body immersion in a warm water (37°C) bath, which donated a large amount of heat. However, whole-body warm water immersion is contra-indicated because it increases the risk of cardiovascular collapse in hypothermic people (Zafren et al., 2014). Notably, in this study, brief T_w_ rewarming did not alter the larger T_re_ after-drop in mildly hypothermic subjects compared with those subjected to spontaneous rewarming in T_a_. Therefore, the results of any study involving mildly hypothermic subjects should be interpreted cautiously with respect to the situation of severe hypothermia (Hoskin et al., 1986). The anatomical differences between men and women (Stephenson and Kolka, 1993; Janssen et al., 2000; van Marken Lichtenbelt et al., 2009), and weakened thermoregulatory system response to extreme temperature conditions in older people (Kenney and Munce, 2003) suggest that the results of the present study may not be directly applicable to women, children, or older people. Water immersion is known to increase venous return and hence stroke volume (Schmid et al., 2007). However, we failed to find any other study which has investigated how these changes in pressure due to water immersion affect cognition, motor drive and perception. These responses in our study were out of scope and thus not evaluated.

## Conclusion

We examined whether brief rewarming in warm water is sufficient to modify hypothermia-induced central and peripheral changes. Our primary finding was that brief (5-min) whole-body rewarming in T_w_ (37°C) rather than rewarming in T_a_ (24°C) for the same duration blunted hypothermia-induced alterations in sensation, motor drive, and cognition despite similarly lower T_re_ and deep T_mu_ in whole-body rewarming in T_w_ (37°C) and rewarming in T_a_ (24°C). We have offered one possible explanation for our findings, but further research is needed to draw firm conclusions, especially regarding the brief post-rewarming effects on neurophysiological and cognitive functional kinetics until full body temperature recovery using other external heating methods that are more suitable for field situations.

## Author contributions

MB designed the studies; HP and NE performed the experiments; HB and LD provided assistance; MB, AS, HP, LD, and NE analyzed and interpreted data; MB and HP wrote the manuscript.

### Conflict of interest statement

The authors declare that the research was conducted in the absence of any commercial or financial relationships that could be construed as a potential conflict of interest.
